# Tissue and imaging biomarkers for hypoxia predict poor outcome in endometrial cancer

**DOI:** 10.18632/oncotarget.12004

**Published:** 2016-09-13

**Authors:** Anna Berg, Kristine E. Fasmer, Karen K. Mauland, Sigmund Ytre-Hauge, Erling A. Hoivik, Jenny A. Husby, Ingvild L. Tangen, Jone Trovik, Mari K. Halle, Kathrine Woie, Line Bjørge, Atle Bjørnerud, Helga B. Salvesen, Werner Henrica M. J., Camilla Krakstad, Ingfrid S. Haldorsen

**Affiliations:** ^1^ Center for Cancer Biomarkers, Department of Clinical Science, University of Bergen, Norway; ^2^ Department of Gynecology and Obstetrics, Haukeland University Hospital, Norway; ^3^ Department of Radiology, Haukeland University Hospital, Norway; ^4^ Section of Radiology, Department of Clinical Medicine, University of Bergen, Norway; ^5^ Department of Physics, University of Oslo, Norway; ^6^ The Intervention Center, Oslo University Hospital, Norway; ^7^ Center for Cancer Biomarkers, Department of Biomedicine, University of Bergen, Norway

**Keywords:** endometrial carcinoma, endometrial hyperplasia, HIF-1α, MRI, FDG-PET/CT

## Abstract

Hypoxia is frequent in solid tumors and linked to aggressive phenotypes and therapy resistance. We explored expression patterns of the proposed hypoxia marker HIF-1α in endometrial cancer (EC) and investigate whether preoperative functional imaging parameters are associated with tumor hypoxia. Expression of HIF-1α was explored both in the epithelial and the stromal tumor component. We found that low epithelial HIF-1α and high stromal HIF-1α expression were significantly associated with reduced disease specific survival in EC. Only stromal HIF-1α had independent prognostic value in Cox regression analysis. High stromal HIF-1α protein expression was rare in the premalignant lesions of complex atypical hyperplasia but increased significantly to invasive cancer. High stromal HIF-1α expression was correlated with overexpression of important genes downstream from HIF-1α, i.e. *VEGFA* and *SLC2A1* (*GLUT1*). Detecting hypoxic tumors with preoperative functional imaging might have therapeutic benefits. We found that high stromal HIF-1α expression associated with high total lesion glycolysis (TLG) at PET/CT. High expression of a gene signature linked to hypoxia also correlated with low tumor blood flow at DCE-MRI and increased metabolism measured by FDG-PET. PI3K pathway inhibitors were identified as potential therapeutic compounds in patients with lesions overexpressing this gene signature. In conclusion, we show that high stromal HIF-1α expression predicts reduced survival in EC and is associated with increased tumor metabolism at FDG-PET/CT. Importantly; we demonstrate a correlation between tissue and imaging biomarkers reflecting hypoxia, and also possible treatment targets for selected patients.

## INTRODUCTION

Endometrial cancer (EC) is the most common gynecologic carcinoma in western countries, and the incidence is increasing [1]. Similar to other solid tumors, hypoxia is an aggressive feature in EC, and may also be used for selecting patients for treatments targeting biological changes related to hypoxia [2–4]. Hypoxia-inducible factor (HIF) proteins orchestrate the expression of numerous genes important for cell adaption to hypoxia. Both in cell line and clinical studies HIF-target genes have been demonstrated to encode proteins necessary for angiogenesis, metabolism, epithelial-to-mesenchymal transition (EMT), invasion, metastasis, stem cell maintenance, immune evasion and response to therapy [5]. HIF-1α is primarily regulated post-transcriptionally through proteasome degradation when oxygen is available, and is recognized as a marker of hypoxia [6]. When the oxygen level drops, HIF-1α is stabilized and translocated to the nucleus from where it, together with HIF-1β, serves as a transcription-promoting factor [6]. HIF-1α is recognized as a marker of poor survival in many solid tumors. In EC the prognostic importance of HIF-1α protein is not established, likely due to lack of consensus in immunohistochemical evaluation of tumor protein expression [7, 8]. For EC, also when considering different cellular localization of HIF-1α (nuclear vs cytoplasm) in the tumor cells, results are conflicting [9–12]. Recently the important function of the hypoxic tumor microenvironment in driving tumor progression and in the development of therapy resistance has been acknowledged, and in the tumor stroma HIF-1α has a key role which has led increased focus in clinical research to discover new therapeutics inhibiting this protein or its targets [8, 13–15]. Also, in EC, stromal protein expression signatures have been shown to be correlated to aggressive clinicopathological features, and also, gene expression in tumor cells to be correlated to expression of downstream proteins in the stroma, indicating an important role of the microenvironment in endometrial carcinogenesis [16].

Advanced imaging techniques using dynamic contrast-enhanced magnetic resonance imaging (DCE-MRI) and positron emission tomography/computed tomography (PET/CT) enable visualization and quantification of functional tumor characteristics *in vivo*. In uterine cancers, these imaging techniques have been employed to depict alterations in tumor microstructure, microcirculation and metabolism relevant for clinical phenotype and prognosis, and some of these imaging findings have a putative link to tumor hypoxia [2, 17–19]. In EC, low tumor blood flow on DCE-MRI is associated with reduced progression free survival [18], and increased microvascular proliferation, a marker of activated angiogenesis, which may also be related to tumor hypoxia [2]. Similarly, in cervical cancer low values for the DCE-MRI parameter A_Brix_ were reported to be linked to a hypoxic tumor phenotype, demonstrated by upregulation of hypoxia response genes and increased HIF-1α protein expression [17]. The important roles of HIF-1α and hypoxia in therapy resistance have been found to be reversed by inhibiting or deleting HIF-1α [20–23]. Hence, understanding the biologic mechanisms involved in tumor hypoxia is therefore important to develop novel targeted treatment strategies and to select the patients most likely to respond to this treatment. Hence, if functional imaging methods are able to depict and quantify changes in tumor hypoxia during therapy, this potentially provides early non-invasive predictive markers of response to hypoxia targeting therapies.

In the present study we explore HIF-1α expression in EC in relation to preoperative functional imaging markers reflecting tumor microvasculature and metabolism, and find these parameters to be linked. Additionally, we investigate to what extent markers of hypoxia in tumor tissue and from preoperative imaging are reflected in a clinical phenotype. Finally, we discuss how this could assist in the optimization and individualization of EC treatment.

## RESULTS

827 patients with comprehensive clinical data available were included in this population based and prospective patient series. Endometrial tissue from hysterectomy specimen were collected and routinely investigated by pathologist at Haukeland University Hospital and classified as complex atypical hyperplasia (CAH) (*n* = 80), endometrioid EC (*n* = 612) and non-endometrioid EC (*n* = 135). A subset of 282 patients had fresh frozen tissue available for RNA extraction and gene expression analysis. Also overlapping with the clinical annotated patient series, 164 had been subject to preoperative DCE-MRI and 108 had preoperative FDG-PET/CT.

### Stromal HIF-1α expression is an independent prognostic marker in endometrial carcinomas

**Figure 1:**
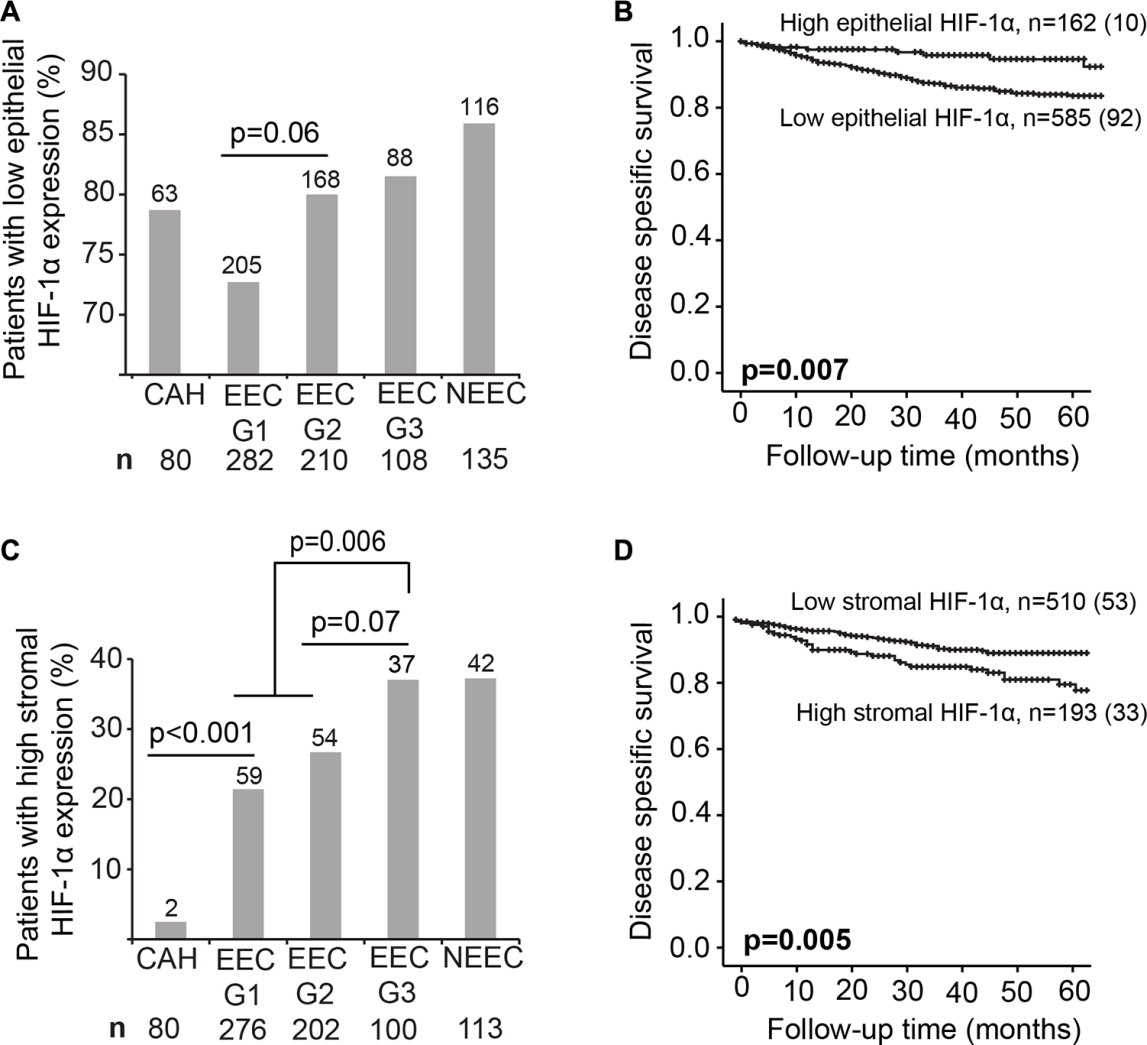
HIF-1α protein expression is a prognostic marker (**A** and **C**) Percentage of patients with low epithelial and high stromal HIF-1α expression in complex atypical hyperplasia (CAH), endometrioid endometrial carcinoma (EEC) grade 1, 2 and 3 and non-endometrioid subtype (NEEC). Number of patients indicated above the bars. Total number of patients indicated under the graph. Given *p*-values are calculated between the indicated groups using Chi-squared test. (**B** and **D**) Kaplan-Meier curves depicting disease specific survival in endometrial cancer (EC) patients according to high or low HIF-1α epithelial (B) and stromal (D) protein expression. For each category: number of cases (number of disease specific deaths).

We investigated expression patterns of HIF-1α in both the stromal and the epithelial tumor component in EC. Low tumor epithelial HIF-1α protein expression was more often observed in patients with non-endometrioid subtype, high histological grade, deep myometrial invasion, lymph node metastasis, high International Federation of Gynecology and Obstetrics (FIGO) stage and in tumors with loss of hormone receptors (Table 1, Figure 1A). Low epithelial HIF-1α expression was significantly associated with reduced disease specific survival in EC (*n* = 747, *p* = 0.007) (Figure 1B). On the other hand, high HIF-1α expression in the tumor stroma was significantly more frequent in patients with non-endometrioid tumor subtype, high grade tumors, lymph node metastasis, high FIGO stage and in tumors with loss of hormone receptors (Table 2, Figure 1C). In addition, high expression of HIF-1α in the tumor stroma was significantly associated with reduced survival (*p* = 0.005) (Figure 1D) and tend to be more frequent in lesions with low epithelial HIF-1α expression (*p* = 0.24, data not shown). Full sections of a small subset of patients (*n* = 29) were explored for epithelial and stromal HIF-1α expression in correlation to high through-put analysis of TMAs. We found protein expression score in full sections to correlate well with expression scores from TMAs for both epithelial and stroma HIF-1α (*p* = 0.04 and *p* = 0.003, respectively) (Supplementary Table 1). Importantly, high stromal HIF-1α expression remained an independent unfavorable prognostic factor (hazard ratio (HR) of 1.7, *p* = 0.04) in a Cox proportional hazards model, when adjusted for epithelial HIF-1α expression, patient age, histological type and grade, myometrial invasion and lymph node metastasis (Table 3), epithelial HIF-1α expression failed to reach statistical in the same multivariate analysis (*p* = 0.97). When including hormone receptor status in the multivariate analysis, neither HIF-1α stromal protein expression nor epithelial HIF-1α expression were significant prognostic markers. Notably, in patients with hormone receptor expression loss, high stromal HIF-1α protein expression is associated with decreased survival (*p* = 0.01), and there was also a tendency in multivariate analysis (*p* = 0.07). Interestingly, high HIF-1α expression in stromal cells was almost non-existing in premalignant lesions, but was frequently observed in low grade endometrioid EC lesions (*p* < 0.001) (Figure 1C).

**Table 1:** Association between epithelial HIF-1α expression and clinicopathological phenotype and hormone receptor status in endometrial cancer patients

Variable	Categories	Epithelial HIF-1α protein expression		
		Low % (*N*)	High % (*N*)	P*
Histological type & grade	EEC Grade 1–2	76 (373)	24 (119)	0.03
*N* = 735	EEC Grade 3	82 (88)	18 (20)	
	NEEC	86 (116)	14 (19)	
Myometrial infiltration	< 50%	75 (339)	25 (114)	0.002
*N* =732	> 50%	85 (236)	15 (43)	
Lymph node metastasis	No	76 (406)	24 (126)	0.004
*N*=596	Yes	92 (59)	8 (5)	
FIGO stage (2009)	I/II	76 (473)	24 (147)	0.001
*N* = 735	III/IV	90 (104)	10 (11)	
ERα protein expression	High	76 (412)	24 (128)	0.01
*N* = 727	Low	85 (159)	15 (28)	
PR protein expression	High	76 (426)	24 (133)	0.005
*N* = 727	Low	86 (145)	14 (23)	

Abbreviations: HIF, hypoxia-inducible factor; EEC, endometrioid endometrial cancer; NEEC, non-endometrioid endometrial cancer; ER, estrogen receptor; PR; progesterone receptor; N, number of patients assessed for epithelial HIF-1α protein expression.

* Chi-square test.

**Table 2:** Association between stromal HIF-1α expression and clinicopathological phenotype and hormone receptor status in endometrial cancer patients

Variable	Categories	Stromal HIF-1α protein expression		
		High % (*N*)	Low % (*N*)	P*
Histological type & grade	EEC Grade 1–2	24 (113)	76 (365)	0.001
*N* = 691	EEC Grade 3	37 (37)	63 (63)	
	NEEC	37 (42)	63 (71)	
Myometrial infiltration	< 50%	27 (116)	73 (317)	0.5
*N* = 688	> 50%	29 (75)	71 (180)	
Lymph node metastasis	No	29 (144)	71 (360)	0.03
*N* = 558	Yes	43 (23)	57 (31)	
FIGO stage (2009)	I/II	26 (153)	74 (438)	0.007
*N* = 691	III/IV	39 (39)	61 (61)	
ERα protein expression	High	23 (121)	77 (405)	< 0.001
*N* = 684	Low	43 (68)	57 (90)	
PR protein expression	High	23 (128)	77 (417)	< 0.001
*N* = 683	Low	44 (61)	56 (77)	

Abbreviations: HIF, hypoxia-inducible factor; EEC, endometrioid endometrial cancer; NEEC, non-endometrioid endometrial cancer; ER, estrogen receptor; PR; progesterone receptor; N, number of patients assessed for stromal HIF-1α protein expression.

* Chi-square test.

**Table 3:** Prognostic value of epithelial and stromal protein expression of HIF-1α in relation to established prognostic factors in endometrial cancer

Variable	*N* (%)	Unadjusted^a^			Adjusted		
		HR	95% CI	P*	HR	95% CI	P*
Age at treatment (mean=64.1)	557	1.1	1.0–1.1	< 0.001	1.0	1.0–1.1	0.009
Histological type & grade							
Endometrioid Grade 1–2	383 (68.8)	1			1		
Endometrioid Grade 3	79 (14.2)	6.6	3.1–13.6	< 0.001	4.8	2.2–10.0	< 0.001
Non-Endometrioid	95 (17.1)	16.0	8.3–30.9	< 0.001	10.8	5.4–21.9	< 0.001
Myometrial invasion				< 0.001			0.006
< 50%	351 (63.0)	1			1		
> 50%	206 (37.0)	3.8	2.3–6.4		2.3	1.3–4.1	
Lymph node metastasis				< 0.001			< 0.001
No	503 (90.3)	1			1		
Yes	54 (9.7)	8.0	4.8–13.3		3.0	1.7–5.4	
Epithelial HIF-1α				0.03			0.970
Low	434 (77.9)	2.5	1.1–5.6		1	0.4–2.5	
High	123 (22.1)	1			1.0		
Stromal HIF-1α				0.004			0.04
Low	391 (70.2)	1			1		
High	166 (29.8)	2.1	1.3–3.4		1.7	1.0–2.9	

HIF, hypoxia-inducible factor; CI, confidence interval; HR, hazard ratio; N, number of cases.

a Unadjusted (univariate) analysis conducted in 557 patients with data available for all variables in the adjusted (multivariate; including all listed variables) analysis.

* Cox proportional hazard model.

### Stromal HIF-1α expression correlates to gene sets representing inflammation and cell cycle regulation and to high metabolic tumor activity measured by FDG-PET/CT

We were puzzled by the strong prognostic significance of stromal HIF-1α protein expression, and the observed absence of stromal expression in premalignant lesions. We therefore explored gene expression alterations in tumor tissue in relation to high stromal HIF-1α protein expression. The genes *VEGFA* and *SLC2A1* are regulated by HIF-1α and strongly linked to angiogenesis and glycolysis, respectively. We found gene expression value of *VEGFA* and *SLC2A1* in the tumor component to be significantly positively correlated to stromal HIF-1α (Supplementary Figure 1). We did not find any difference in expression of *HIF-1*α mRNA in relation to histological type and grade (Supplementary Figure 2A), while mRNA expression levels of *VEGFA* and *SLC2A1* were significantly higher in the more aggressive histologic phenotypes (Supplementary Figure 2B–2C). To further explore molecular alterations in premalignant and malignant tumors with high stromal HIF-1α, we performed a Gene Set Enrichment Analysis (GSEA). Using Gene Ontology (GO) annotated gene sets supplied by the Broad Institute; inflammation and cell cycle regulation were identified as activated in lesions with high stromal HIF-1α expression as compared to lesions with low stromal HIF-1α expression (Supplementary Table 2).

**Figure 2:**
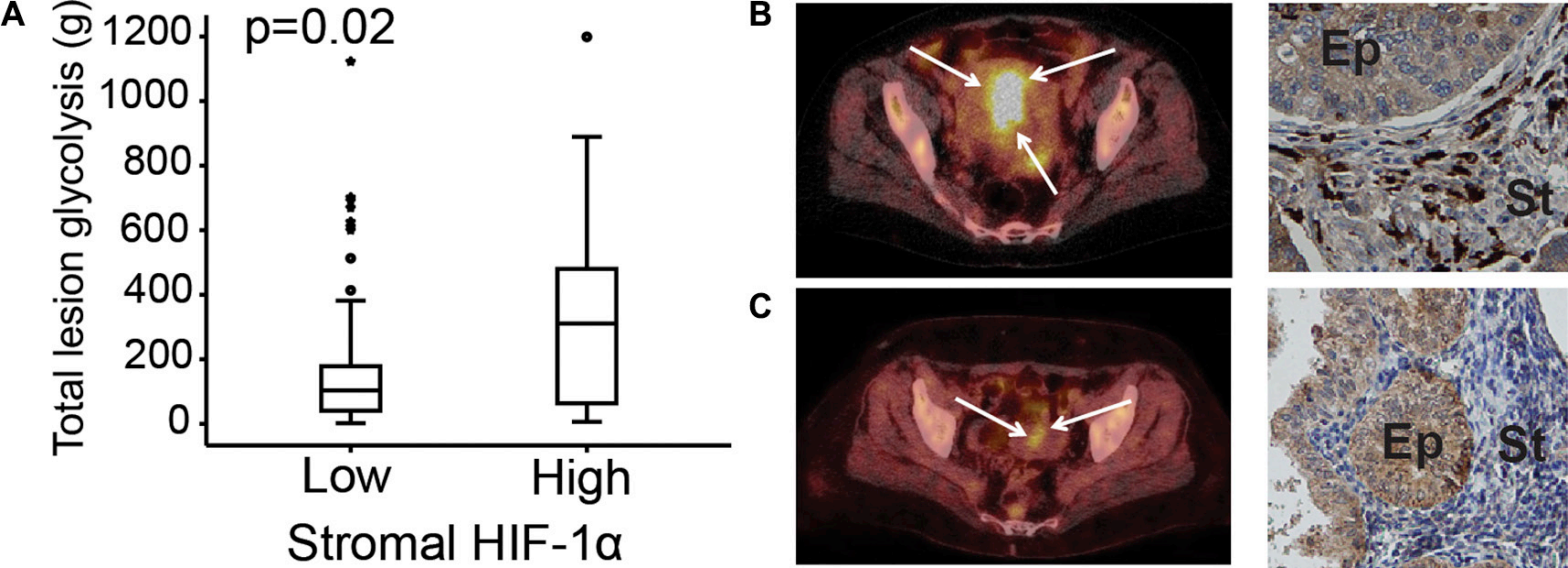
Stromal HIF-1α protein expression correlated to 18FDG PET/CT markers Total lesion glycolysis (TLG) assessed by FDG-PET/CT in primary endometrial carcinoma lesions compared to stromal HIF-1α protein expression (**A**). FDG-PET/CT and HIF-1α expression in a 77-year-old patient with a non-endometrioid endometrial cancer (NEEC), FIGO stage 1B: PET/CT exhibits a large FDG-avid uterine tumor (white arrows) with high TLG and the tumor had high stromal HIF-1α expression (black arrows) (**B**) FDG-PET/CT and HIF-1α expression in a 58-year-old patient with endometrioid endometrial cancer (EEC), grade 2, FIGO stage 1A: PET/CT exhibits a small uterine lesion (white arrows) with low TLG and the tumor had low stromal HIF-1α expression (black arrows) (**C**). Ep: epithelial tumor compartment. St: stromal tumor compartment.

The FDG-PET parameters total lesion glycolysis (TLG) was significantly higher in tumors with high stromal HIF-1α expression (*n* = 99, *p* = 0.02; Table 4 and Figure 2). Furthermore, high stromal HIF-1α expression tended to be associated with higher tumor maximum and mean standardized uptake value (SUV_max_, SUV_mean_) and metabolic tumor volume (MTV) (*p* ≤ 0.17 for all; Table 4). No significant associations were observed between the metabolic PET parameters and epithelial HIF-1α expression or between the functional MRI parameters and both expression categories of HIF-1α expression (Table 4). However, high tumor volume estimated by MRI tended to be associated with high stromal HIF-1α expression (*p* = 0.21) (Table 4).

**Table 4:** Tumor HIF-1α protein expression pattern in relation to preoperative functional MRI and metabolic FDG-PET/CT parameters of endometrial cancer lesions

Imaging parameter	Epithelial HIF-1α			Stromal HIF-1α		
	Low Mean (*N*)	High Mean (*N*)	P*	High Mean (*N*)	Low Mean (*N*)	P*
**MRI**						
Fb (ml/100 ml/min)	49.7 (124)	48.0 (37)	0.99	47.5 (48)	50.6 (109)	0.67
K^trans^ (/min)	0.037 (122)	0.034 (37)	0.64	0.038 (48)	0.035 (107)	0.27
kep (/min)	0.42 (122)	0.44 (36)	0.29	0.44 (48)	0.42 (106)	0.22
V_e_ (ml/100 ml)	11.4 (122)	9.1 (36)	0.23	11.7 (48)	10.4 (106)	0.59
IAUGC (mMs)	69 (124)	68 (37)	0.81	69 (48)	69 (109)	0.80
ADC (× 10^−6^ m^2^/s)	800 (123)	789 (36)	0.50	787 (48)	802 (107)	0.64
Volume (ml)	22 (124)	15 (37)	0.48	30 (48)	16 (109)	0.21
**FDG PET-CT**						
SUV_max_ (g/ml)	14.8 (75)	14.1 (27)	0.79	16.0 (29)	14.0 (71)	0.17
SUV_mean_ (g/ml)	5.9 (75)	5.8 (26)	0.76	6.5 (28)	5.6 (71)	0.06
MTV (ml)	33 (77)	24 (29)	0.13	45 (30)	25 (74)	0.09
TLG (g)	235 (75)	169 (26)	0.43	336 (28)	173 (71)	**0.02**

Abbreviations: HIF, hypoxia-inducible factor; MRI, magnetic resonance imaging; FDG-PET/CT, fluorodeoxyglucose positron emission tomography/computed tomography; N, number of cases; Fb, blood flow; K^trans^, transfer from blood to EES; kep, transfer constant from blood extravascular extracellular space (EES) to blood; V_e_, fractional volume of EES; IAUGC, integrated area under the concentration time curve; ADC, apparent diffusion coefficient; SUV_max_, maximum standard uptake value; SUV_mean_, mean standard uptake value; MTV, metabolic tumor volume; TLG, total lesion glycolysis.

* Mann-Whitney *U*-test.

### Hypoxia gene signature score is associated with tumor mRNA expression of related genes and functional imaging parameters and suggests treatment targets

**Figure 3:**
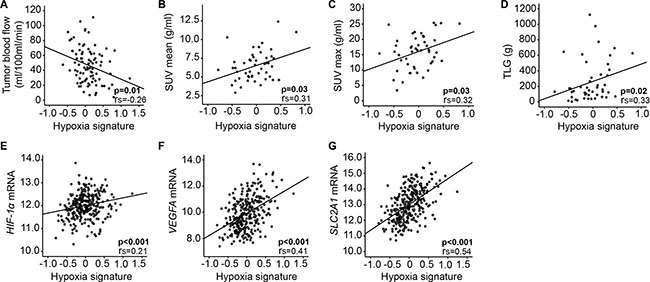
Hypoxia gene signature correlates to imaging and tissue biomarkers A pre-defined hypoxia gene signature [17] correlates to DCE-MRI parameter in 91 primary endometrial cancer (EC) lesions (**A**), FDG-PET/CT in 48 primary EC lesions (**B**–**D**) and expression of relevant genes (**E**–**G**) in 282 patients with complex atypical hyperplasia (CAH, *n* = 22) and primary endometrial cancer (EC, *n* = 260). r_s_ = correlation coefficient.

When applying a published hypoxia gene signature based on DCE-MRI in cervical cancer [17] to our EC dataset, the hypoxia gene signature score was significantly anti-correlated to tumor blood flow (*p* = 0.01) (Figure 3A). Also, hypoxia signature score was correlated to tumor volume (*p* = 0.05) and tended to be correlated to mean apparent diffusion coefficient (ADC), representing cellular density (*p* = 0.07) (data not shown). Furthermore, SUV_mean_, SUV_max_ and TLG were positively correlated with expression of the same hypoxia gene signature score (*p* ≤ 0.03 for all) (Figure 3B–3D), and so were *HIF-1α*, *VEGFA* and *SLC2A1* mRNA expression (*p* < 0.001 for all) (Figure 3E–3G). High stromal HIF-1α protein expression also tended to be correlated to this hypoxia gene signature (*p* = 0.06, data not shown).

We queried connectivity map (CMap) (version 2) [24] for compounds anti-correlated to gene expression in lesions expressing high hypoxia signature score versus low hypoxia signature score, to identify drugs with potential to treat patients with hypoxic tumors. The PI3K inhibitor LY294002 emerged as top-ranked along with the HDAC inhibitor Trichostatin A and the HSP90 inhibitor Tanespimycin (*p* < 0.001 for all) (Supplementary Table 3).

In our patient cohort with available gene expression data, 83 of 282 patients had received adjuvant chemo- or radiotherapy. Consistent with previous findings [22], radiotherapy treated patients (*n* = 25) with high hypoxia signature score (*n* = 20) tended to have decreased overall survival (*p* = 0.16) (data not shown).

### Cancer associated fibroblasts express HIF-1α in endometrial cancer lesions

**Figure 4:**
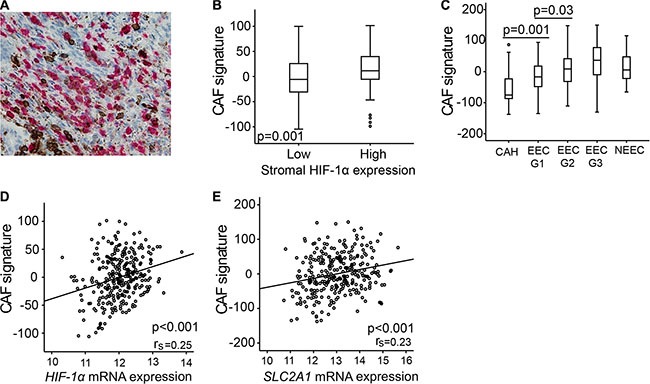
Carcinoma associated fibroblasts (CAFs) gene signature overexpressed in lesions of high stromal HIF-1α expression (**A**) Immunohistochemical double staining of CD45 (brown) and HIF-1α (red) simultaneously, picture demonstrating few double stained cells (40× magnification). (**B**) CAF gene signature according to high or low stromal HIF-1α protein expression. (**C**) CAF signature score in patients with complex atypical hyperplasia (CAH), endometrioid endometrial cancer (EEC) grade 1, 2 and 3, and non-endometrioid endometrial cancer (NEEC). (**D**) CAF signature score correlated to *HIF-1α* mRNA level. (**E**) CAF signature score correlated to *SLC2A1 (GLUT1)* mRNA level. r_s_ = correlation coefficient.

The histological appearance of the stromal cells with high HIF-1α protein expression, including irregular branched cytoplasm, suggested these to be cancer associated fibroblasts (CAFs). There are numerous protein markers expressed by CAFs, however none are uniquely expressed in these cells [25]. We therefore performed simultaneously IHC staining for HIF-1α and common leukocyte antibody (CD45) in a subset of tumor samples (*n* = 95), to exclude that the HIF-1α expressing cells in the stroma are tumor associated macrophages (TAMs) or tumor infiltrating lymphocytes (TILs). Despite some leucocytes and occasional granulocytes staining positive for CD45, most of the HIF-1α positive cells did not express CD45 (Figure 4A). Furthermore, a gene signature derived from breast cancer exhibiting different gene expression patterns in CAFs and fibroblasts from non-cancerous tissue [26], demonstrated a significantly increased signature score in endometrial lesions with high stromal HIF-1α expression (*p* = 0.001) (Figure 4B). This CAF signature was significantly higher in low grade endometrioid cancer than in CAH (*p* = 0.001) and was also positively correlated to *HIF-1α* and *SLC2A1* mRNA expression (*p* < 0.001) (Figure 4C–4E).

## DISCUSSION

In this large population based study of EC patients, we demonstrate the negative prognostic impact of high stromal and low epithelial HIF-1α expression. Furthermore, imaging markers of increased tumor metabolism and low tumor blood flow are associated with overexpression of stromal HIF-1α and a hypoxia gene signature. Our findings support that tumor hypoxia may be important in EC progression, and reveal that functional preoperative imaging by DCE-MRI and FDG-PET/CT could depict and quantify functional alterations relevant for tumor hypoxia in EC.

We show that stromal and epithelial HIF-1α expression tend to be inversely correlated, and that high stromal HIF-1α expression and low epithelial HIF-1α expression is associated with reduced survival. Similar to our findings, stromal hypoxia (represented by increased HIF-2α and downstream proteins) has been shown to be a negative prognostic factor in colorectal cancer, whereas epithelial tumor protein expression had no prognostic significance [27]. Hypoxia is frequently observed in solid tumors [15] and is believed to promote tumor cells to become more invasive and aggressive [28]. Even though hypoxia is a dynamic process in tumor tissue, multiple protein markers have been demonstrated to represent this biological state. HIF-1α is one of the most important, in addition to HIF-2α, carbonic anhydrase (CA) IX, glucose transporter (GLUT) 1 and osteopontin (OPN) [29]. HIF-2α and HIF-1α are structurally similar; however, HIF-2α is thought to be less important in EC, with detected HIF-2α expression in less than 20% of tumor cells [30]. Literature evaluating the prognostic relevance of HIF-1α protein expression patterns in EC is conflicting. Studies exploring protein expression in both nucleus and cytoplasm of the tumor cells have demonstrated HIF-1α to be a negative prognostic factor both in uni- and multivariate survival analysis [9]; while others have failed to find a correlation with survival [10]. Also in studies exploring only the nuclear expression, the prognostic value of HIF-1α is not established [11, 12]. The inverse prognostic impact of stromal and epithelial HIF-1α protein expression we observe, may explain some of the discrepancies in reported prognostic impact of HIF-1α in the literature. Our approach, assessing HIF-1α protein expression patterns separately in the epithelial cells and the stromal cells has, to our knowledge, not previously been described in EC.

Our finding of high stromal HIF-1α expression in primary cancers and very low expression in premalignant lesions suggests that HIF-1α positive cells in the tumor stroma are important in early steps of endometrial carcinogenesis. Histological characteristic of these cells points towards fibroblasts; this is also the most abundant stroma cell type [31]. In our patient cohort HIF-1α expressing cells in the stroma do generally not co-express CD45; indicating that stromal HIF-1α positive cells are not immune derived (TILs and TAMs). The CAF gene signature identified to be overexpressed in lesions overexpressing stromal HIF-1α supports this. The important role of tumor fibroblasts in tumor initiation and progression is well-established [32, 33]. CAFs have recently been recognized as important drivers of tumor growth and angiogenesis, and also crucial for the metabolic shift to glycolysis [25, 34, 35]. In line with this, the CAF gene signature is strongly linked to *HIF-1α* and *SLC2A1* mRNA level, and correlates to early invasive phenotype (endometrioid EC grade 1 and 2). The tumor microenvironment is increasingly recognized as an important contributor to cancer development and progression [36], and the genetic stability of the cells comprising the stroma makes it an attractive target for therapy [31]. The cells in the tumor stroma, including tumor infiltrating lymphocytes (TILs), tumor associated macrophages (TAMs) and cancer associated fibroblasts (CAFs) have been demonstrated to play different roles in tumor development and progression under the influence of hypoxia and HIF-1α [3, 6, 25, 37]. CAFs, the most abundant cell type in the tumor stroma, have been demonstrated to have an important role in cancer initiation and the metastatic step, and also CAFs are induced by HIF-1α and a key source of VEGF, which is essential in tumor angiogenesis [25, 31].

We show that the metabolic tumor marker TLG is significantly positively correlated to stromal HIF-1α protein expression. This is also true for gene expression of *SLC2A1*, indicating that energy production by glycolysis (aerobic or anaerobic) is the preferred pathway in these tumors [35]. Concurrently, SUV_max_, SUV_mean_ and MTV, by FDG-PET/CT tend to be higher in lesions overexpressing stromal HIF-1α. A positive correlation between tumor HIF-1α protein expression and tumor FDG uptake has also been shown in squamous cell carcinoma of the head and neck [38, 39] and in breast cancer [40], but to our knowledge not previously in EC. In line with the metabolic shift we observe in tumors overexpressing stromal HIF-1α, we find that a hypoxia gene signature (based on DCE-MRI derived parameters in cervical cancer) [17] is associated with low tumor blood flow (Fb) and high tumor metabolism (SUV_max_, SUV_mean_ and TLG), further suggesting a link between these imaging markers and tumor hypoxia. The positive correlation between the same hypoxia gene signature and *HIF-1α*, *VEGFA* and *SLC2A1* suggests that the imaging parameters reveal real functional aspects reflecting the oxygen status in the tumor. Importantly, our CMap query indicates well-known anti-neoplastic substances like LY294002 (a PI3K inhibitor), Trichostatin A (a HDAC inhibitor) and Tanespimycin (a HSP90 inhibitor) as promising potential treatment options for patients with increased expression of the hypoxia gene signature. Also, these drugs reportedly down-regulate HIF-1α and sensitize tumors for radiation therapy [41, 42]. In endometrial cancer cell lines inhibition of the PI3K pathway is demonstrated to sensitize cancer cells to radiotherapy through down regulation of HIF-1α [13]. Additionally, direct suppression of HIF-1α increased cell death after radiation, indicating that combined PI3K- and HIF-1α inhibition might potentiate the treatment effect [13]. Furthermore, blood flow, assessed by MRI is anti-correlated to this hypoxia gene signature, and could represent a non-invasive method of selecting patients for these treatment options, however in our cohort with gene expression data; too few patients have received adjuvant therapy to address this question.

For the subset of patients with preoperative DCE-MRI we did not find any significant correlations between MRI derived structural (ADC, volume) or functional (Fb, K^trans^, kep, V_e_ and IAUGC) tumor parameters and the expression of stromal or epithelial HIF-1α. This contrasts a previous study in EC, reporting an association between low blood flow (Fb) at preoperative DCE-MRI and increased tumor microvascular proliferation, putatively linked to tumor hypoxia. Thus, although Fb has been linked to increased microvascular proliferation in EC, it is not proven to be the best marker of hypoxia [2].

We find stromal HIF-1α expression to be highly significantly associate with *SLC2A1* mRNA expression where the corresponding protein, GLUT1, is known as an important protein facilitating increased glycolysis on which the cancer cells and hypoxic cells are dependent [36]. Additionally, stromal HIF-1α expression is highly significantly correlated to *VEGFA* mRNA expression. HIF-1α orchestrates downstream genes essential to the cells' adaption to hypoxia [37], and HIF-1α is an important transcription inducer for numerous growth factors and cytokines in tumor angiogenesis, most importantly *VEGFA* [43]. In this respect, the hypoxic influence of the stroma has been suggested to be more important than the effect on the tumor cells [37]. Inhibition of HIF-1α has been shown to increase the effect of radiotherapy, proposed to be due to the selective killing of hypoxic cells, which are not sensitive to the DNA damaging effect of radiotherapy [22]. Other mechanisms that might be involved are reduced mitochondrial oxygen consumption, normalization of microvasculature, as well as loss of stromal ability to adapt to post-radiation tumor ischemia [21, 22]. Also, hypoxia and HIF-1α have been shown to induce epithelial-to-mesenchymal-transition (EMT), increasing the metastatic potential of carcinomas [28].

In conclusion, high stromal HIF-1α is associated with an aggressive histological subtype and with reduced disease specific survival in EC. Lesions with high stromal HIF-1α expression exhibited increased tumor metabolism at FDG-PET/CT, and functional PET/CT and DCE-MRI parameters were correlated to previously published hypoxia gene signature scores. Our findings support that tissue markers of hypoxia are reflected in clinical phenotype and associated with functional imaging markers in EC. These preoperative imaging markers, in combination with molecular tissue markers, seem promising and should be further studied to guide selection of patients for novel treatment algorithms targeting hypoxia in EC.

## MATERIALS AND METHODS

### Patient and tissue samples

Patients surgically treated for EC or CAH at Haukeland University Hospital between May 2001 and January 2015 were included in the study. Written informed consent was obtained from all patients for the collection of imaging data and specimens for biomarker studies included in an institutional review board-approved protocol (Rek Vest 2009/2315).

Tumor samples were obtained from hysterectomy specimens and collected in the Bergen Gynecologic Cancer Biobank, and linked to clinical and histopathological data. In total, 827 patients with primary EC (*n* = 747) and CAH (*n* = 80) with available IHC staining for HIF-1α were included. Imaging of patients with primary EC was implemented in the preoperative protocol for patients with primary EC from June 2009 for DCE-MRI and from June 2011 for FDG-PET/CT. 164 and 108 patients with available tissue for protein expression had undergone preoperative DCE-MRI and FDG-PET/CT, respectively. A subset of 22 CAH and 260 EC patients had fresh frozen tissue used for RNA extraction and mRNA analysis. Clinical information included age at primary treatment (in primary EC) or diagnosis (in CAH), body mass index (BMI), menopausal status, parity, primary surgical treatment and additional therapy received. For patients with EC, FIGO stage, histologic subtype, grade and follow-up data were recorded.

Written informed consent was obtained from all patients for the collection of imaging data and specimens for biomarker studies included in an institutional review board-approved protocol (Rek Vest 2009/2315).

### Magnetic resonance imaging protocol and derived imaging parameters

Preoperative magnetic resonance imaging (MRI) was conducted on a whole-body 1.5-T MRI system (Siemens Avanto running Syngo v. B17, Erlangen, Germany) using a six-channel body coil applying a standardized imaging protocol [18]. To reduce motion artefacts 20 mg butylscopolamine bromide (Buscopan; Boehringer, Ingelheim, Germany) was administered intravenously just prior to scanning. Mean/median (range) interval between MRI examination and surgery was 12/9 (1–98) days. Structural MRI included pelvic sagittal and axial oblique (perpendicular to the long axis of the uterus) T2-weighted images and axial oblique T1-weighted gradient-echo images. T1-weighted series were acquired before and after intravenous administration of gadoterate meglumine (Dotarem, Guerbet: 0.1 mmol gadolinium per kilogram of body weight, 3 ml/s injection speed) using a 2- min delay. Pelvic diffusion weighted imaging (DWI) was acquired using an axial two-dimensional echo planar imaging (EPI) sequence with b-values of 0 and 1000 s/mm^2^ with calculation of ADC maps. Pelvic DCE-MRI was acquired with 12 axial slices using a three-dimensional (3D) spoiled gradient echo (FLASH) sequence with a temporal resolution of 2.49 s [18] and a total of 160 dynamic scans.

Tumor ADC values were extracted in regions of interest (ROIs) which were manually drawn on the ADC maps in a representative part of the tumor, using the slice depicting the largest cross-sectional tumor area. Tumor ROI was also drawn on the DCE images at 2 min post-contrast on the slice with the largest cross-sectional tumor area. Parametric maps were generated from the DCE series based on the extended Tofts kinetic model, as well as model free deconvolution using singular value decomposition for blood flow estimates [44], using a standardized arterial input function reported in the literature [45]. The following DCE-MRI tumor parameters were calculated: blood flow (Fb), transfer constant from extravascular extracellular space (EES) to blood (kep), transfer from blood to EES (K^trans^), volume of EES (V_e_) and integrated area under the concentration time curve (IAUGC). Tumor volume was estimated by measuring the largest tumor diameter in three orthogonal planes (a, b, c) using the following equation: Tumor volume = a × b × c/2 [46].

### FDG-PET/CT and derived imaging parameters

PET/CT was performed on a Biograph 40 True Point scanner (Siemens). The scanning covered from the caput to the proximal thigh. The protocol included 6 h of fasting before the imaging was conducted. 18F-FDG (322–414 MBq) was given intravenously 60–120 min before the CT scan. Low-dose CT (120 kV, 50 mAs) for attenuation correction of the PET data was executed before the static emissions, which were obtained at intervals of 3 min per bed position; subsequently, intravenous contrast agent (Iomerol, 350 mg iodine/mL; Bracco Imaging Scandinavia, AB) and negative oral contrast agent (water) were administered for the diagnostic CT scan (120 mV, 240 mAs).

The PET images were fused with both the diagnostic and the low-dose CT images and the metabolic tumor measurements were performed using the low-dose fusion images. Mean/median (range) interval between PET-scanning and primary treatment was 15/12 (1–102) days. The SUV_max_ was recorded, and MTV and SUV_mean_ were measured in a volume of interest (VOI) including voxels with an SUV of more than 2.5. TLG in the tumor was also estimated using the following equation: TLG = SUV_mean_ × MTV [19].

### Oligonucleotide DNA microarray analyses

RNA was extracted from fresh frozen tumor tissue after selection of area of high tumor purity, according to the procedure as previously described [47]. The RNA was hybridized to Agilent Whole Human Genome Microarrays according to manufacturer's instruction (www.agilent.com). The arrays were subsequently scanned by the Agilent Microarray Scanner Bundle. Signal intensity was interpreted using the software J-Express. The majority of samples had tumor purity above 80% with a minimum threshold > 50%. The patient series with RNA microarray was overlapping with protein expression scores for epithelial HIF-1α in 250 patients (18 cases with CAH) and stromal HIF-1α in 238 patients (18 cases with CAH).

Transcriptional alterations were explored by GSEA [48]. Pre-defined gene sets supplied by the MSigDB (www.broadinstitute.org/gsea/index.jsp) were used to compare gene sets with strongest differential expression between two groups. Hypoxia gene signature score was calculated in similar manner as the in the original paper, taking the average expression of the 31 included genes [17]. High hypoxia signature score was defined as the three upper quartiles, and low as the lowest quartiles, based on the size of the groups and number of events in the quartile groups in a survival analysis. Significance Analysis of Microarrays (SAM) was used to find genes differentially expressed in lesions with high vs low hypoxia gene signature score. Genes included in further analysis had a FDR of 0 and a fold change above 2. 201 genes were down-regulated in lesions with high hypoxia signature score, and 44 genes were up-regulated. These gene lists were used to quire the publically available database Connectivity Map (CMap) (www.broadinstitute.org/cmap) [24] for compounds with the potential to reverse the hypoxic tumor state represented by a hypoxia gene signature.

### Immunohistochemical staining

Tissue Micro Arrays (TMAs) were prepared as previously described and included three 0.6 mm cylinders from the most representative tumor area on FFPE tissue blocks from all patients [49]. Immunohistochemistry (IHC) was performed for ERα, PR and HIF-1α in TMA slides as previously reported [50]. Details regarding antibodies and conditions are described in Supplementary Table 4. Hormone receptor negativity was defined as loss of protein expression of both ERα and PR (double negative).

The double staining procedure assessing HIF-1α and CD45 was conducted in a subsample of 95 patients in a similar manner. After microwave antigen retrieval was completed, Dual Endogenous Enzyme Block (Dako S2003) was applied (8 min), followed by Protein Block (Dako X0909) for 10 minutes. The two primary antibodies were added simultaneously and incubated for 60 minutes, followed by washing, and 30 min incubation with secondary antibodies (Goat anti-rabbit, AP (Southern Biotech 4050–04) and EnVision+System-HRP, anti-mouse (Dako K4001). Alkaline Phosphatase chromogen (Liquid permanent red, Dako K0640) was applied, followed by HRP chromogen (DAB^+^). After counterstaining with Hematoxylin the slides were covered by Faramount (Dako S3025) and cover slides.

The stained slides were evaluated using the well-established semi-quantitative staining index graded from 0–9 as a product of staining intensity (0–3) and the area with this intensity (0–3) [49]. All three proteins (ERα, PR and HIF-1α) were assessed in the epithelial compartment of the tumor, and HIF-1α also in the stroma. Established cut-off values were applied as previously reported for ERα and PR [49, 50]. For epithelial and stromal HIF-1α protein expression, high expression was defined as upper quartile, and low expression as the lower three quartiles, based on the number of events in the quartile groups in a survival analysis.

### Statistical analysis

Analyses were performed using the statistical software SPSS (Statistical Package of Social Science) version 23.0. *P*-values reported were two-sided, at a significance level of < 0.05. Differences in continuous data were assessed using a non-parametric Mann-Whitney-*U* test. Pearson-Chi-squared test was used to analyze variances in categorical data. Correlation between epithelial and stromal HIF-1α expression in each patient was explored by McNemar test for paired data. Spearman correlation was used for assessing the association between continuous data. In univariate analysis of disease specific survival the Kaplan-Meier method (log rank test) was used, with date of primary surgical treatment as entry date and the date of death due to EC as endpoint. Cox proportional hazards regression analysis was used for multivariate analyses.

## SUPPLEMENTARY MATERIALS


