# Supplementary material for: Tissue and imaging biomarkers for hypoxia predict poor outcome in endometrial cancer
Source: Oncotarget. 2016 Sep 13;7(43):69844–56. doi: 10.18632/oncotarget.12004 (PMC5342519; doi:10.18632/oncotarget.12004)
Supplement: Supplementary file 1 [file oncotarget-07-69844-s001.pdf]

Tissue and imaging biomarkers for hypoxia predict poor outcome in endometrial cancer

Supplementary Materials

Supplementary Table S1: Evaluation of HIF-1α expression using tissue micro arrays shows a significant overlap with evaluation of full sections

	Full sections				*P*
**TMA**	Epithelial HIF-1α (%)		Stromal HIF-1α (%)		
	Low (*n*)	High (*n*)	Low (*n*)	High (*n*)	
Epithelial HIF-1α: Low	88 (14)	12 (2)			0.04
High	46 (6)*	54 (7)			
Stromal HIF-1α Low			77 (10)	23 (3)	0.003
High			19 (3)	81 (13)	

*Two cases showed heterogeneous expression pattern. Full sections from 29 patients stained for HIF-1α.

Supplementary Table S2: Gene Ontology (GO) annotated gene sets supplied by the Broad Institute (MSigDB).

Rank	Gene set	*P*	FDR (%)
1	M_PHASE	0.0	0.0
2	DEFENSE_RESPONSE	0.0	0.0
3	M_PHASE_OF_MITOTIC_CELL_CYCLE	0.0	0.0
4	MITOSIS	0.0	0.0
5	CELL_CYCLE_PHASE	0.0	0.0
6	CELL_CYCLE_PROCESS	0.0	0.0
7	INFLAMMATORY_RESPONSE	0.0	0.0
8	RESPONSE_TO_OTHER_ORGANISM	0.0	0.0
9	IMMUNE_SYSTEM_PROCESS	0.0	0.0
10	MITOTIC_CELL_CYCLE	0.0	0.0

Top ranked gene sets enriched in endometrial (malignant and premalignant) lesions with high expression of stromal HIF-1α compared to low stromal protein expression.

Supplementary Table S3: Top ranked therapeutic agents anti-correlated to lesions expressing high hypoxia signature score versus low hypoxia signature score using Connectivity Map (version 2)

Rank	Compound	*N*	Enrichment	*P*	Description
1	LY-294002	61	−0,356	0	PI3K inhibitor
2	Trichostatin A	182	−0,22	0	HDAC inhibitor
3	Tanespimycin (17-AAG)	62	−0,27	0,0002	HSP90 inhibitor

Supplementary Table S4: Methods applied for immunohistochemical staining

Primary antibody		Secondary antibody	Vendor	Buffer[d]	Incubation time	Incubation temperature	Dilution
ERα	MM[a]	Anti-mouse HRP (K4001)	Dako (M7047)	pH 9	30 min	Room temp.	1:50
PR	MM[a]	Anti-mouse HRP (K4001)	Dako (M3569)	pH 9	60 min	Room temp.	1:150
HIF-1α	MR[b]	Anti-rabbit HRP (K4002)[c]	Abcam (ab51608)	pH 9	60 min	Room temp.	1:200
CD45	MM[a]	Anti-mouse HRP (K4001)	Dako (M0701)	pH 9	60 min	Room temp.	1:200

[a]Monoclonal mouse, [b]Monoclonal rabbit, [c]When stained together with CD45 anti-Rabbit AP was used (Southern Biotech, 4050-04), [d]Target Retrieval Solution, pH9 (Dako, S2367).

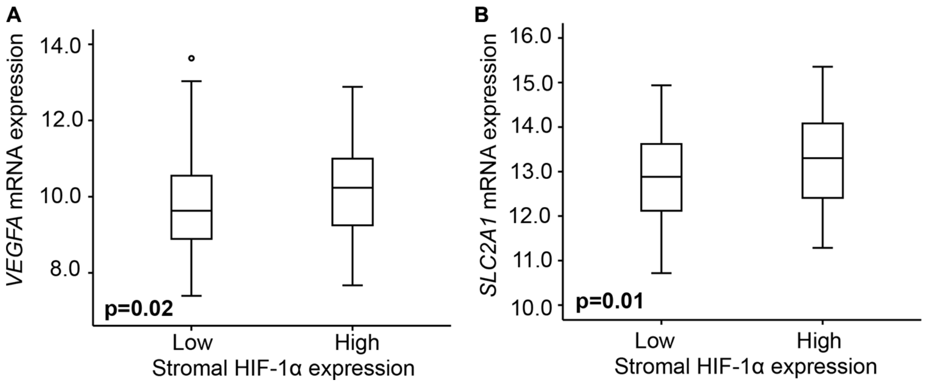

Supplementary Figure S1: Gene expression according to stromal HIF-1α protein expression. Gene expression value of *VEGFA* (**A**) and *SLC2A1* (**B**) in primary endometrial cancer (EC) lesions and complex atypical hyperplasia (CAH) according to high and low stromal HIF-1α protein expression.

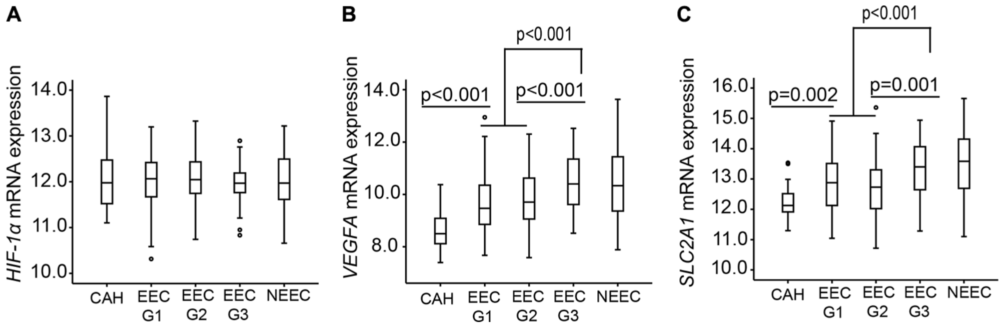

Supplementary Figure S2: Gene expression according to histological type and grade. Gene expressions value of *HIF-1α* (**A**), *VEGFA* (**B**) and *SLC2A1* (**C**) in complex atypical hyperplasia (CAH) patients, endometrioid endometrial cancer (EEC) grade 1, 2 and 3, and non-endometrioid endometrial cancer (NEEC).
